# Glycosylation Profiles in Cardiovascular Diseases: A Bibliometric Analysis

**DOI:** 10.34133/hds.0409

**Published:** 2026-02-03

**Authors:** Tianqi Chang, Jingyu Wang, Chenyu Fan, Yuzhou Xue, Jiaxing Wang, Ming Xu

**Affiliations:** ^1^ Department of Cardiology and Institute of Vascular Medicine, Peking University Third Hospital, State Key Laboratory of Vascular Homeostasis and Remodeling, NHC Key Laboratory of Cardiovascular Molecular Biology and Regulatory Peptides, Beijing Key Laboratory of Cardiovascular Receptors Research, Peking University, Beijing, China.; ^2^ Department of Cardiology, The First Affiliated Hospital, Zhejiang University School of Medicine, Hangzhou, China.; ^3^ Renal Division, Peking University First Hospital, Peking University, Beijing, China.; ^4^ Research Unit of Medical Science Research Management/Basic and Clinical Research of Metabolic Cardiovascular Diseases, Chinese Academy of Medical Sciences, Beijing, China.

## Abstract

**Background:** Cardiovascular diseases (CVDs) continue to be the leading cause of morbidity and mortality globally, indicating a major global health burden. Glycosylation, one of the key posttranslational modifications of proteins, plays an important role in the onset and progression of CVDs. This study employed bibliometric analysis to examine the research on glycosylation and CVDs, aiming to identify the evolution and hotspots in this field. **Methods:** A total of 1,441 publications published from 2010 January 1 to 2024 December 31 were extracted from the Web of Science Core Collection. The analysis included a visual and descriptive examination of publication trends, countries/regions, institutions, keywords, and references. **Results:** The United States is the most productive country/region in this field, followed closely by China. The University of Alabama at Birmingham has made the most important contribution to this area. Key research hotspots include “O-GlcNAcylation”, “biomarkers”, “angiogenesis”, “α-dystroglycan”, “potassium channel”, “heart failure”, “gene expression”, “glycosylation”, and “cardiac glycosides”. **Conclusion:** Research on glycosylation in CVDs has shown a steady increase in recent years. Among these studies, O-GlcNAcylation plays a pivotal role in this field. This comprehensive bibliometric analysis of glycosylation and CVDs provides researchers with valuable, objective insights to support future investigations.

## Introduction

Glycosylation, one of the most abundant posttranslational modifications (PTMs) of proteins, involves the covalent attachment of carbohydrates to proteins. This modification plays a critical role in modulating the structure, stability, and biological activity of proteins, thereby influencing cellular signal transduction, cell adhesion, and receptor activation. Glycosylation includes several covalent linkages to proteins, such as N-linkage to asparagine (N-glycosylation), O-linkage to serine and threonine residues (O-glycosylation), and C-mannosylation, S-glycosylation, and P-glycosylation [1].

Glycosylation, a pivotal PTM, has made remarkable progress in research for clinical diseases in recent years, particularly in cancer [2,3], diabetes [4,5], neurodegenerative diseases [6], and immune disorders [7,8]. Abnormal glycosylation patterns, such as changes in glycosylation sites or structural alterations in glycans, have been shown to be closely associated with the onset, progression, and prognosis of diseases. Aberrant glycosylation patterns have been shown to promote tumor progression by enhancing cell proliferation, facilitating metastasis through altered cell–matrix interactions, and enabling immune evasion via modulation of immune checkpoint ligands and glycan-mediated masking of tumor antigens [9,10]. Because of the effects on protein function and intercellular interactions, glycosylation and associated enzymes have become potential drug targets. The glycosylation structures of antibody drugs, particularly in the treatment of cancer, greatly impact their solubility, stability, and therapeutic efficacy [11,12]. Modulating the glycosylation state of these drugs can enhance their effectiveness or reduce side effects [13,14]. The importance of glycosylation in clinical diseases highlights its potential as a valuable therapeutic target.

Glycosylation plays a pivotal role in both the physiological and pathological processes of the cardiovascular system [15]. Abnormal glycosylation is closely associated with heart failure (HF) [16], cardiac hypertrophy [17], and diabetes-related cardiac injury [18]. Moreover, dysregulated glycosylation can disrupt cell adhesion, exacerbate inflammation, and promote vascular remodeling, ultimately contributing to conditions such as atherosclerosis [19,20]. Therefore, a systematic and comprehensive examination of scientific publications on glycosylation in cardiovascular diseases (CVDs) is essential to deepen our understanding of current research. While several reviews have summarized the progress in glycosylation and CVDs, there remains a lack of objective, data-driven visualizations, with a heavy reliance on researchers’ subjective interpretations. The subjectivity and heterogeneity limit our ability to comprehensively evaluate the current research and effectively determine future directions in the field.

This study employed bibliometric analysis to evaluate publication trends, keyword evolution, and highly cited references from 2010 to 2024. The comprehensive bibliometric visualization aims to provide an objective overview of the current status hotspots of glycosylation in CVDs, offering valuable insights for researchers. The study provides an objective overview of the research landscape of glycosylation in CVDs, analyzes the evolution of this field, and highlights emerging topics, ultimately promoting further research in glycosylation and CVDs.

## Materials and Methods

### Data source

The rationale for selecting the Science Citation Index Expanded (SCIE) subset of the Web of Science Core Collection for bibliometric analysis is elaborated in detail in our previous work [21–23]. SCIE, as a flagship citation index, ensures that the quality control of journals and related publications reaches a high standard. The journals included in SCIE undergo rigorous peer-review processes, and they enjoy widely recognized credibility in terms of academic normativity, rigor of research methods, and the impact of research outcomes. This effectively avoids interference with bibliometric analysis results caused by the inclusion of low-quality or nonacademic literature [24]. Furthermore, as one of the most comprehensive and authoritative citation databases worldwide covering fields such as natural sciences and engineering technologies, SCIE not only provides standardized literature cataloging formats and complete citation network information but also ensures the comparability and compatibility of research results from different countries and institutions. This standardization feature is crucial for cross-regional and cross-temporal bibliometric analyses, as it can provide a consistent and reliable data foundation for revealing the knowledge structure, hotspots of collaborative networks, and evolutionary trends in research fields [23,25]. In summary, this approach maximizes the capture of core publications while minimizing potential omissions.

### Search strategy, data extraction, cleaning, and analysis

The final search strategy is as follows: (TS= (“Protein Glycosylation” OR glycosylation OR “N-glycosylation” OR “O-glycosylation” OR “O-GlcNAcylation” OR “C-mannosylation” OR “S-glycosylation” OR “P-glycosylation”)) AND (TS= (cardiac OR cardiovascular OR coronary OR heart OR vascular)) [26] NOT (TS= (“Non-enzymatic glycosylation” OR glycation).

**Fig. 1.**
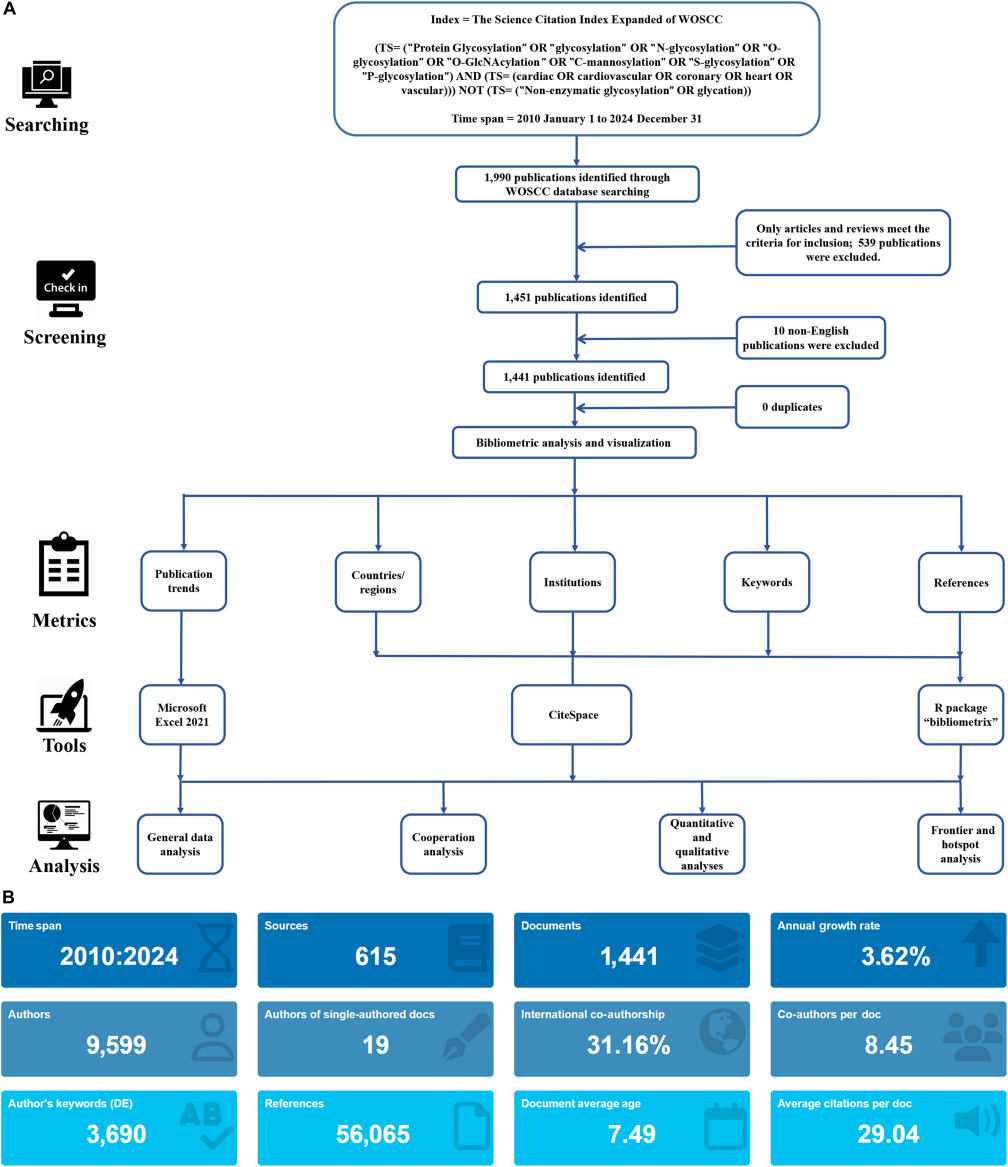
The procedures for the bibliometric analysis. (A) Flowchart of the bibliometric analysis on glycosylation and cardiovascular diseases (CVDs). (B) Additional statistics on glycosylation publications in CVDs from R bibliometrix. WOSCC, Web of Science Core Collection; DE, document elements.

As shown in Fig. 1, the procedures and standards for data extraction, downloading, and processing remain consistent with those outlined previously [27–30]. Data were extracted on 2024 December 31, and CiteSpace analysis confirmed no duplicate publications.

When processed with CiteSpace, the time slicing was set at 1-year intervals, and *κ* was assigned a value of 35 to ensure reasonable clustering under this *κ* value. No simplification or “pruning” was performed on the processing of all knowledge structures. This was intended to fully preserve all associative relationships between knowledge units in the original data, avoiding the loss of any potential connection and interaction information that might hold research value. In doing so, the study could accurately capture even the relatively hidden developmental trajectories and associative patterns within the knowledge system. The cosine algorithm was adopted for calculating link strength. This algorithm measures the tightness of associations between knowledge units by computing the cosine value of the angle between vectors, which can effectively reflect the similarity and association intensity of different knowledge elements across dimensions such as semantics and co-occurrence. This further ensures the accurate quantification of node connection relationships in the knowledge network, rendering subsequent analyses of the tightness of knowledge associations more scientific and reliable and facilitating a more refined interpretation of the internal structure and dynamic associations of the knowledge network. During the clustering process, keywords were extracted from publications, and the maximum likelihood ratio algorithm was employed to extract and optimize clustering labels [31]. Based on the distribution probability of keywords across different documents, this algorithm accurately identifies the most representative clustering topic labels, effectively distinguishing the core thematic scopes of different knowledge clusters. As a result, the research topic corresponding to each cluster becomes clearer and more explicit, providing precise thematic identifiers for the subsequent clear presentation of research focuses of each knowledge cluster and the sorting out of thematic associations and evolutionary relationships among different clusters. This further enhances the interpretability and research value of the knowledge network clustering analysis results.

In the network visualization generated by CiteSpace [32,33], node size reflects frequency or co-occurrence attributes, while node color represents either the average or the first appearance time. Connections between nodes indicate collaboration or degree of intersection. A key advantage of this network visualization is its ability to highlight pivotal and influential nodes. Betweenness centrality (BC) is a commonly used metric in bibliometric analysis, designed to measure the frequency with which a node appears on the shortest paths between other nodes. Nodes with a BC value greater than 0.1 occupy a central position within the visualization and are considered key and core elements of the network. The *Q*-score is used to evaluate network structure, with values greater than 0.3 indicating a well-structured network. The *S*-score assesses network credibility, with values above 0.7 signifying high reliability. Timeline graphs and burst detection tests are used to visualize the evolutionary trajectories of specific parameters and identify emerging hotspots/frontiers.

### Statistics

Data were visualized and analyzed using CiteSpace (6.4.R1, 64-bit, advanced version) and R 4.3.1 with the “bibliometrix” package. Descriptive statistics, combined with qualitative analysis, were employed to depict the bibliometric landscape.

## Results

### Temporal distribution of publications

**Fig. 2.**
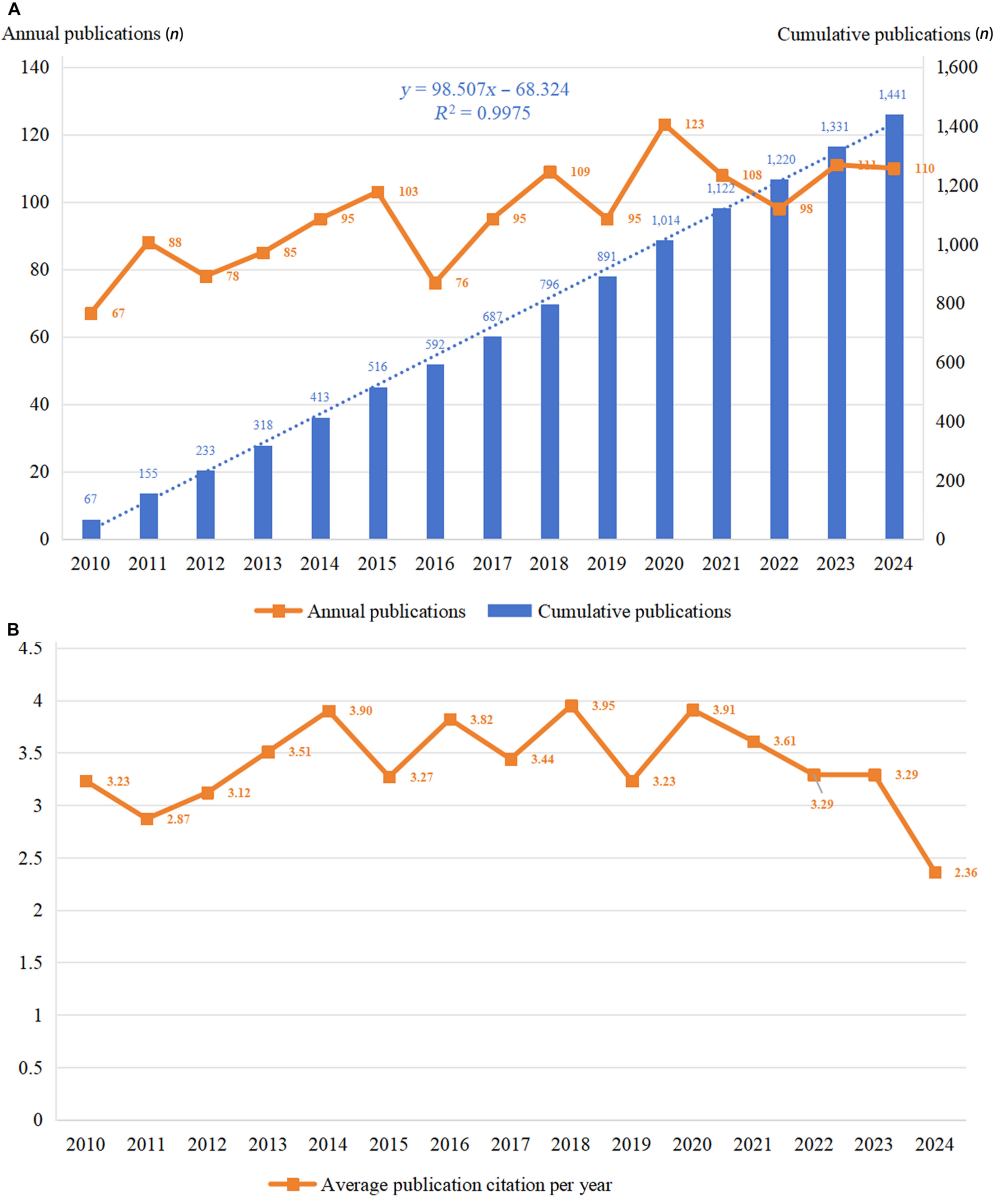
Output and citation of the publications on glycosylation and CVDs. (A) Annual and cumulative global output of research publications on glycosylation and CVDs from 2010 to 2024. (B) Average number of citations per publication per year from 2010 to 2024.

A total of 1,441 publications related to glycosylation and CVDs were retrieved from 2010 January 1 to 2024 December 31. The number of studies on glycosylation and CVDs increased from 67 publications in 2010 to 1,441 publications in 2024 (Fig. 2A). The annual publication volume was around 100 papers, and the annual growth rate was 3.62%. From 2010 to 2015, the number of publications showed a slow upward trend. Between 2016 and 2024, there was a notable fluctuation in publication volume. According to the fitted curve, it is predicted that approximately 1,606 publications in this field will be published in 2026. For subsequent triennial analysis, the volume of publications exhibited a consistent upward trend (Fig. S1). Each publication received 3.39 citations per year on average, accompanied by observable interannual fluctuations in citation frequency (Fig. 2B). Notably, the average annual citations per publication show a marked decline in 2024, which can be attributed to the natural time lag between publication, readership, and subsequent citation in academic research.

### Countries/regions and institutions

**Fig. 3.**
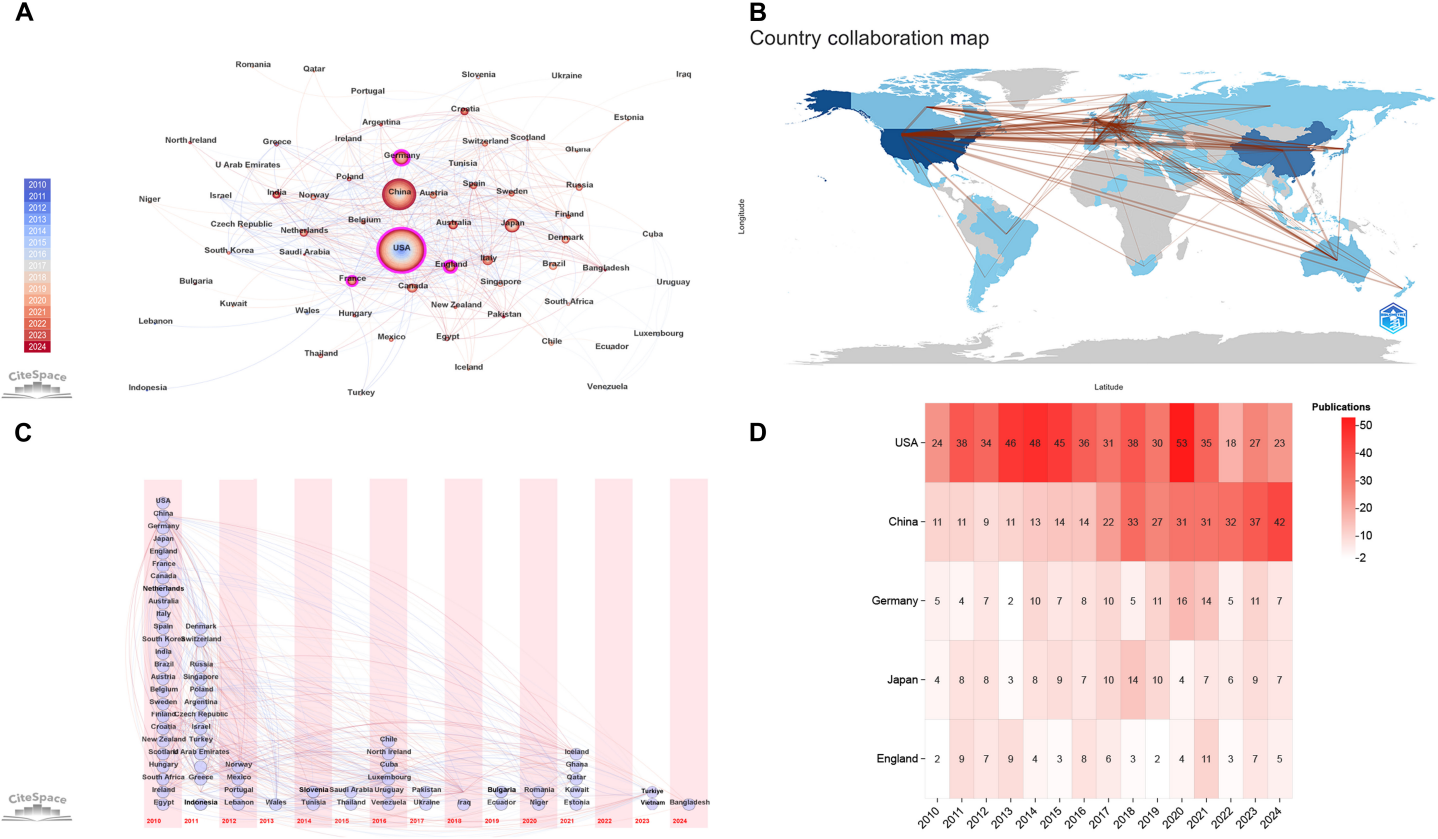
Countries/regions in research for glycosylation and CVDs. (A) A network visualization map of country/region connections, revealing 501 links across 72 countries/regions worldwide. (B) Cross-harmonization plot of countries/regions. (C) Countries/regions entering the field per year from 2010 to 2024. (D) A heat map showing annual publications for the 5 most prolific countries/regions from 2010 to 2024.

Research on glycosylation in CVDs has been conducted in 71 countries/regions (Fig. 3A). Table 1 lists the top 10 countries/regions by publication volume, with the United States of America (USA; *n* = 526), China (*n* = 338), and Germany (*n* = 122) ranking as the top 3, which is consistent with the triennial analysis (Fig. S2a). In terms of the average citation per publication, Canada, France, USA exhibit the highest impact. Figure 3B presents the collaborations and linkages between different countries/regions in research for glycosylation and CVDs. As shown in Fig. 3C, the top 10 countries/regions in research on glycosylation and CVDs were among the earliest to enter the field. A notable increase in the number of countries/regions entering the field was observed in 2016 and 2021, predominantly involving nations with moderate population sizes, ranging from small to medium. Meanwhile, China showed a marked upward trend in its annual publication output, likely attributable to the targeted national health priorities and advancements of glycoproteomics (Fig. 3D).

**Table 1.** The top 10 productive countries/regions in glycosylation and CVDs by publication volume

Ranking	Country/region	Count	Percentage	BC	H-index	AC/P
1	USA	526	34.97%	0.36	73	39.87
2	China	338	22.47%	0.05	40	19.53
3	Germany	122	8.11%	0.21	34	34.31
4	Japan	114	7.58%	0.03	27	22.19
5	England	83	5.52%	0.28	26	28.11
6	France	68	4.52%	0.16	29	44.60
7	Canada	67	4.45%	0.03	28	46.27
8	Netherlands	67	4.45%	0.05	28	38.62
9	Australia	60	3.99%	0.03	22	26.57
10	Italy	59	3.92%	0.02	22	33.18

BC, betweenness centrality; AC/P, average citation per publication

**Fig. 4.**
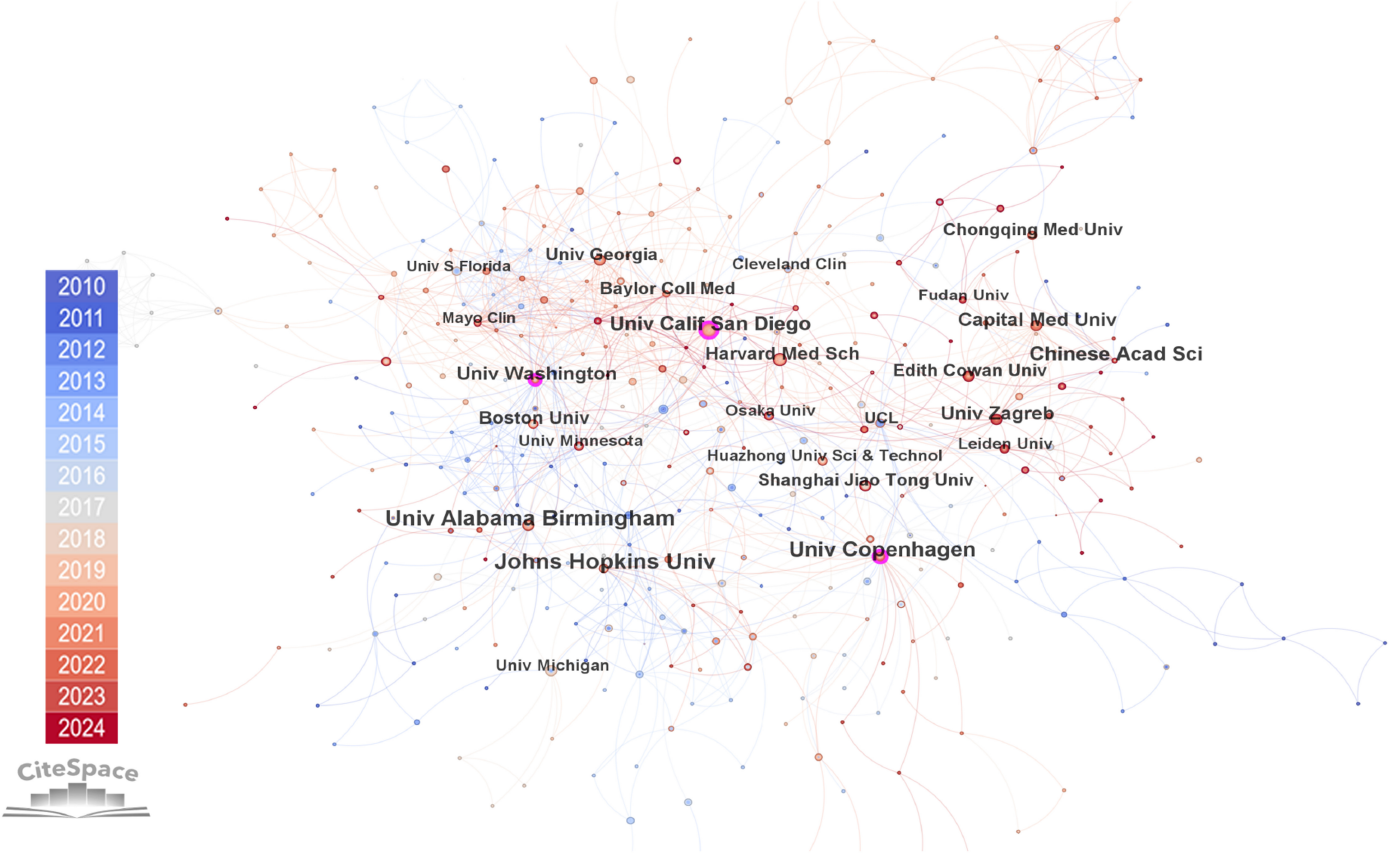
A visual network of 612 institutions worldwide in research for glycosylation and CVDs using CiteSpace visualization.

We extracted 612 institutions that participated in research on glycosylation and CVDs through CiteSpace visualization (Fig. 4). Table 2 lists the top 10 institutions based on publications, with half of them located in the USA. The University of Alabama at Birmingham led with the most publications, contributing 31 papers, followed by Johns Hopkins University (29 publications) and the University of Copenhagen (24 publications). The Chinese Academy of Sciences and Capital Medical University each contributed 18 publications, revealing the substantial involvement of Chinese academic institutions in the research output.

**Table 2.** The top 10 productive institutions in glycosylation and CVDs by publication volume

Ranking	Institution	Count	Percentage	BC
1	University of Alabama at Birmingham	31	14.76%	0.10
2	Johns Hopkins University	29	13.81%	0.08
3	University of Copenhagen	24	11.43%	0.15
4	University of California, San Diego	23	10.95%	0.14
5	University of Zagreb	19	9.05%	0.06
6	Chinese Academy of Sciences	18	8.57%	0.01
7	Capital Medical University	18	8.57%	0.04
8	University of Washington	18	8.57%	0.11
9	Harvard Medical School	16	7.62%	0.05
10	Edith Cowan University	14	6.67%	0.00

### Keyword analysis and burst evaluation

**Fig. 5.**
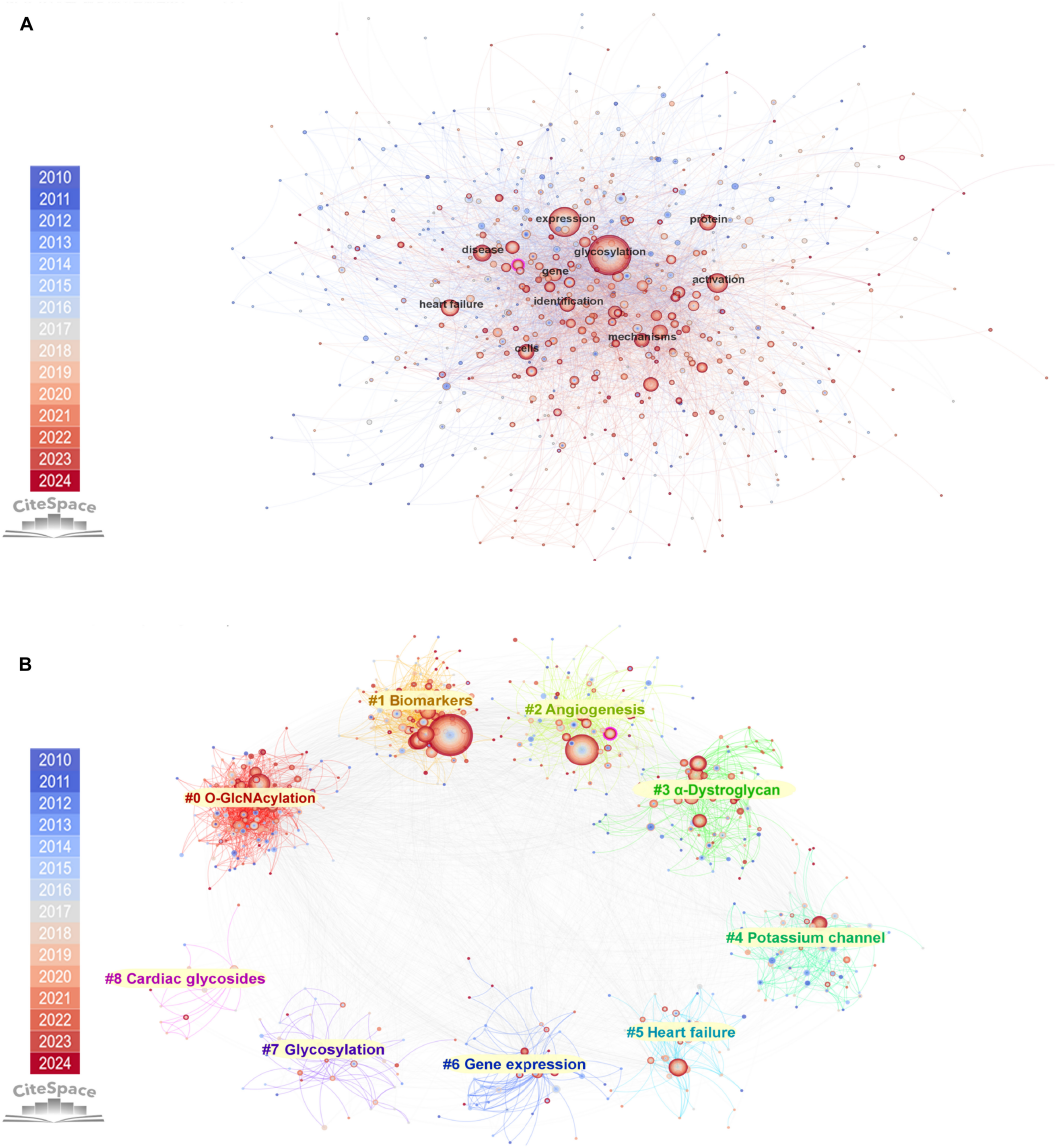
Keywords in the research on glycosylation in CVDs. (A) A visual network of 615 keywords and relevant links using CiteSpace visualization. (B) The clustering map of 615 keywords in research on glycosylation and CVDs.

**Fig. 6.**
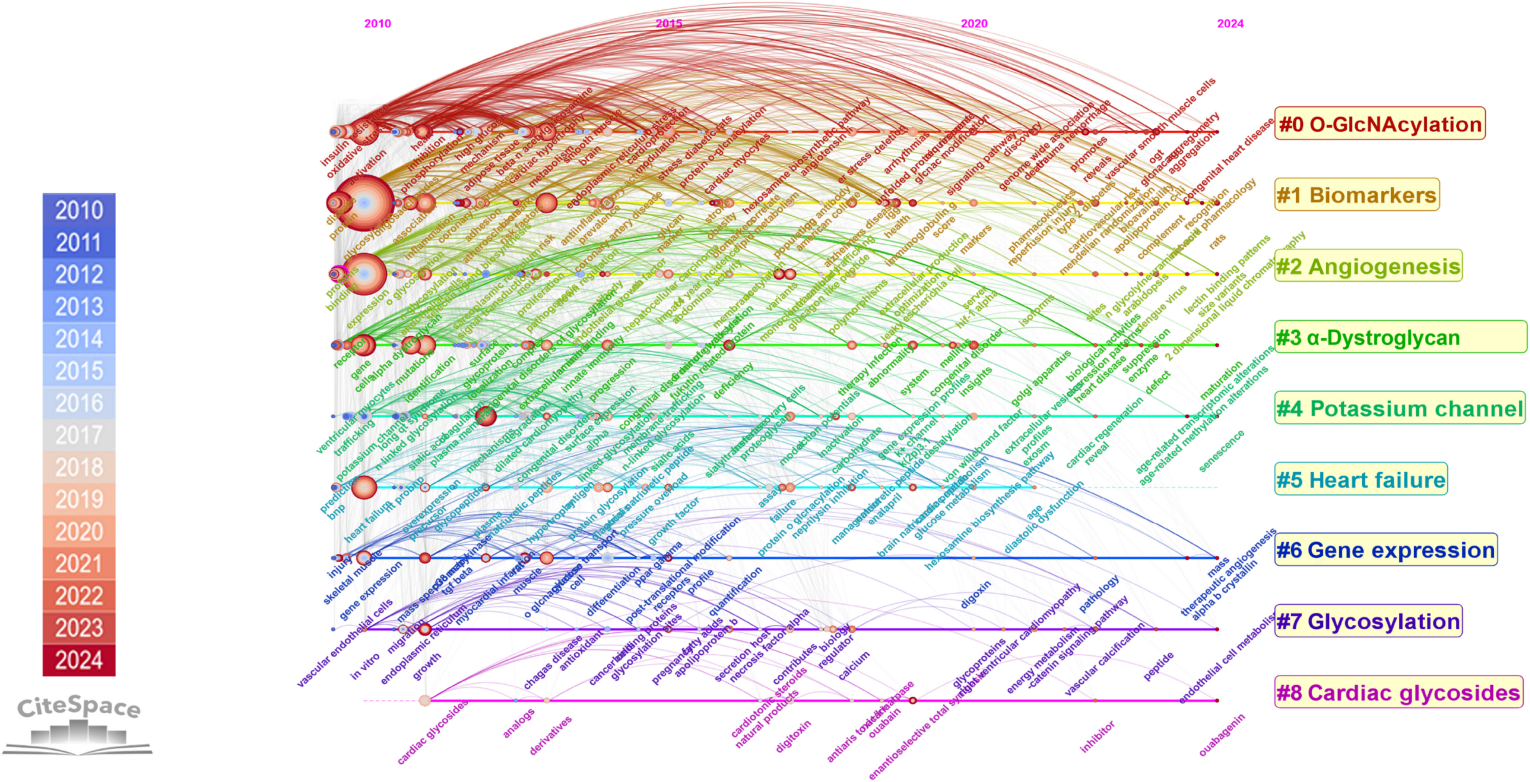
The timeline visualization map for keywords clustering in glycosylation and CVDs.

To understand the core content and further direction of glycosylation in CVDs, we conducted a comprehensive and in-depth analysis of the keywords in the research; 615 keywords were identified (Fig. 5A). Table 3 shows the top 10 keywords, in which the term “glycosylation” has been mentioned 389 times and ranked the first, followed by “expression” (249 times) and “activation” (121 times), aligning with triennial analysis (Fig. S3a). To further figure out the relevant topics in glycosylation and CVDs, 615 keywords were categorized into 9 clusters. The *Q*-score of the clustering map is 0.3589 and the *S*-score is 0.7226, indicating that the result is credible. The categories include “O-GlcNAcylation”, “biomarkers”, “angiogenesis”, “α-dystroglycan”, “potassium channel”, “heart failure”, “gene expression”, “glycosylation”, and “cardiac glycosides” (Fig. 5B). According to the cluster categories and keyword timeline, it is evident that the primary clusters mainly emerged from 2010 to 2015, while the research on glycosylation and CVDs has consistently primarily focused on “O-GlcNAcylation” and “biomarkers” (Fig. 6). For further triennial analysis, the following categories were added: “atherosclerosis”, “identification”, “expression”, “endoplasmic reticulum”, “trafficking”, “susceptibility”, and “escherichia coli”, reflecting broader and more sustained research themes (Fig. S3b).

**Table 3.** The top 10 most used keywords in the research of glycosylation and CVDs

Ranking	Keywords	Count	BC	Year
1	Glycosylation	389	0.04	2010
2	Expression	249	0.03	2010
3	Activation	121	0.06	2010
4	Protein	109	0.03	2010
5	Identification	102	0.04	2011
6	Heart failure	89	0.04	2010
7	Cells	84	0.06	2010
8	Disease	80	0.07	2010
9	Mechanisms	76	0.07	2012
10	Gene	74	0.04	2010

**Fig. 7.**
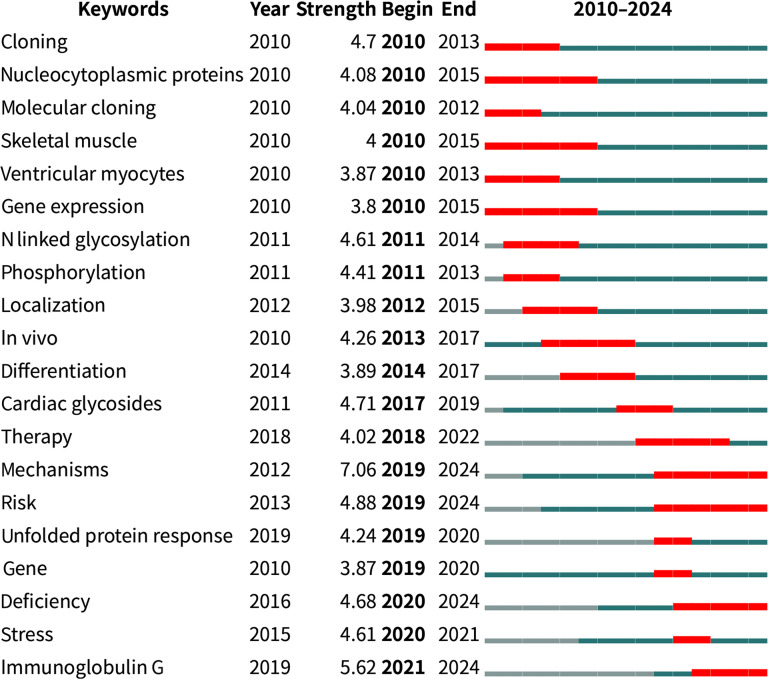
Top 20 keywords with the strongest citation bursts in the research on glycosylation and CVDs.

Burst detection was conducted on the keywords of cited publications for the identification of the study trends and frontiers. The top 20 strongest keywords are presented in Fig. 7. The term “mechanisms” exhibited the highest intensity, with a surge beginning in 2012 and sustaining in 2024. The burst time of “mechanisms”, “risk”, “deficiency”, and “immunoglobulin g” extended to 2024, indicating the sustained attention and growing interest in these areas.

### References

**Fig. 8.**
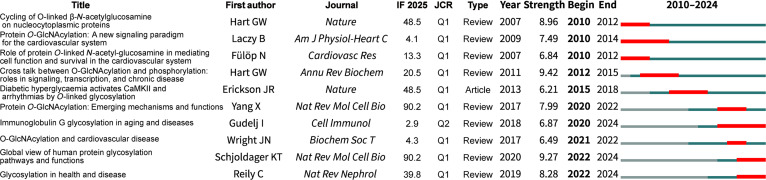
Top 10 references with the strongest citation bursts cited in the research on glycosylation and CVDs. IF, impact factor; JCR, Journal Citation Reports.

CiteSpace identified 926 references in the research on glycosylation in CVDs, and there are 2 crucial references in the collaborative network with a BC value greater than 0.1 (Fig. S4), which are both review articles and focus on O-GlcNAcylation. Citation bursts were evaluated in the 926 references, with the top 10 presented in Fig. 8. Among these references, 90% are review articles, and 70% are related to O-GlcNAcylation. The reference with the strongest burst reveals the relationship between O-GlcNAcylation and phosphorylation [34]. The same author contributed an additional review titled “Cycling of O-linked β-*N*-acetylglucosamine on nucleocytoplasmic proteins” [35]. The sole original research article investigates the effect of O-GlcNAcylation on arrhythmias [36].

## Discussion

### Comprehensive analysis of general information

In 2016, there was a noticeable decline in the number of annual publications related to glycosylation and CVDs, which may be attributed to the interplay of macroeconomic research policies, global public health events, and the developmental stage of relevant technologies. From a policy perspective, 2016 marked the launch of China’s 13th Five-Year Plan (2016 to 2020), a period of strategic realignment in national research funding, which likely caused temporary delays in project approvals and resource allocation for interdisciplinary fields like glycosylation and CVD research. Meanwhile, the marked increase in publication output in 2020 coincides with the final phase of China’s 13th Five-Year Plan, reflecting the potential impact of policy-driven support for strategic scientific and technological advancement. Globally, the Zika virus outbreak, declared a public health emergency of international concern in 2016 [37], shifted biomedical research priorities and resources toward infectious disease control, diverting attention and funding from noncommunicable disease studies, including CVD-related glycosylation research. Additionally, mass spectrometry technologies, critical to glycoproteomics, were still evolving. While mass spectrometry began to be applied in biopharmaceutical glycosylation quality control [38], their widespread adoption in fundamental CVD research remained limited in 2016. Together, these factors contributed to a transient dip in research output, underscoring the profound influence of external events and technological transitions on scientific publication trends. However, from a macro perspective with 3-year intervals, the triennial publication volume in this field shows a consistent upward trend (Fig. S1). Therefore, the decline observed in 2016 is more likely attributable to a temporary fluctuation in the research process rather than being indicative of a reversal in the overall long-term trend.

In the field of glycosylation and CVD research, the shifting publication trends between China and the USA reflect a profound transformation in the global landscape of scientific production. China’s rapidly increasing publication output is largely driven by top-level strategic initiatives. National plans such as the 13th and 14th Five-Year Programs have prioritized life sciences as key development areas, while talent-recruitment policies like the “Thousand Talents Program” have successfully attracted a substantial number of overseas scholars to return, forming high-caliber research teams. In parallel, China’s large population of CVD patients provides a unique and extensive clinical resource, and sustained government investment in core research infrastructure, such as high-end mass spectrometry platforms, has further contributed to the field’s expansion.

In contrast, the USA, as an early leader in this domain, has seen stabilization or a slight decline in publication volume at a relatively high level, reflecting the natural evolution of a mature research system. Foundational and framework-establishing work, such as elucidating the role of O-GlcNAcylation in cardiac stress responses [34,35], has largely been completed. Consequently, current research efforts are increasingly focused on more complex and nuanced scientific questions, like specific glycosyltransferase functions, which entail longer research cycles and slower output. Additionally, amid intensifying competition for research funding and growing labor shortages, US research priorities have increasingly shifted toward high-return areas such as clinical translation [39], cancer immunotherapy [40], gene editing [41], and artificial-intelligence-driven medical technologies [42], thereby reducing support for traditional research areas like glycosylation and CVDs.

This contrast illustrates not only the strategic divergence between the 2 countries at different stages of research system maturity but also underscores the dynamic balance between foundational research and translational priorities in global knowledge production ecosystem. In essence, China and the USA have formed a phase-based complementarity. China leverages systematic investments for rapid catch-up and scale, while the USA capitalizes on its innovative ecosystem to explore cutting-edge questions and paradigm shifts, collectively advancing the field to new depths.

### Analysis of research keywords

#### O-GlcNAcylation in CVDs

O-GlcNAcylation is the addition of O-linked *N*-acetylglucosamine (*O*-GlcNAc) to protein serine/threonine residues, regulated by *O*-GlcNAc transferase and *O*-GlcNAcase. As a dynamic PTM, it modulates target protein function, activity, subcellular localization, and stability [43]. Elevated *O*-GlcNAc levels link to cardiomyocyte dysfunction [44]; the O-GlcNAcylation of phospholamban inhibits its phosphorylation, impairing sarco/endoplasmic reticulum Ca^2+^ ATPase 2a isoform (SERCA2a) function and myocardial contractility [45], while that of A20 accelerates atherosclerosis via altered ubiquitination/degradation [46].

Diabetes increases cardiomyocyte O-GlcNAcylation [47]: hyperglycemia drives Ca^2+^/calmodulin-dependent kinase II (CaMKII) O-GlcNAcylation (activating it, enhancing sarcoplasmic reticulum Ca^2+^ release, and causing cardiac dysfunction/arrhythmias) [36], and modifies dynamin-related protein 1 (DRP1)/phospholamban (reducing phosphorylation, inducing mitochondrial dysfunction and impaired contractility) [45,48]. High glucose also stimulates cardiac fibroblasts to produce excess collagen (promoting fibrosis) [49], disrupts endothelial nitric oxide synthase activation in endothelial cells [50], and impairs perivascular adipose tissue function in metabolic syndrome [51]. In vascular smooth muscle cells, hyperglycemia-induced O-GlcNAcylation upregulates pro-atherosclerotic thrombospondin-1, which trivalent chromium (Cr^3+^) mitigates by reducing O-GlcNAcylation [52]. These findings highlight O-GlcNAcylation’s pivotal role in CVDs, making it a potential therapeutic target.

#### Immunoglobulin G and glycosylation in CVDs

Immunoglobulin G (IgG)—accounting for 75% of serum immunoglobulins—plays a key immune role and has sustained research attention (citation burst through 2024) for its glycosylation–CVDs links. The IgG heavy chain Fc region carries bi-antennary *N*-glycans at asparagine 297 [53]; glycosylation modulates IgG structure, stability, function, and immunogenicity, with core fucose/galactose/sialic acid deficiency enhancing antibody-dependent cellular cytotoxicity [54,55]. It also impacts antibody half-life, antigen affinity/avidity, Fc/Fcγ receptor signaling, and effector functions [56,57].

Specific sialylation-related IgG *N*-glycans inversely correlate with CVD risk, very low-density lipoprotein/triglyceride levels, and carotid plaque [58]. Altered IgG *N*-glycan profiles are associated with hypertension–diabetes comorbidity (Chinese Muslim/Han populations) [59], predict diabetes/CVDs with high accuracy (surpassing established markers) [60,61], correlate with Kazakh blood pressure (potential hypertension biomarker) [62], and link to cardiometabolic risk factors (Chinese cross-sectional study) [63]. Thus, plasma IgG *N*-glycan profiles hold promise for risk stratification in cardiometabolic disease primary prevention.

#### Analysis of pivotal references

HF, the terminal stage of various CVDs, involves structural and functional remodeling of cardiomyocytes, fibroblasts, endothelial cells, and other cell types [64]. PTMs, particularly glycosylation, play a pivotal regulatory role in this process. Advanced HF is often accompanied by metabolic remodeling—such as enhanced glycolysis—which increases hexosamine biosynthetic pathway flux and subsequently elevates intracellular protein O-GlcNAcylation [65,66]. While acute increases in *O*-GlcNAc levels may protect cardiomyocytes under stress, chronic and dysregulated *O*-GlcNAc modification is deemed detrimental. It modulates the function of key proteins (e.g., kinases, phosphatases, and transcription factors) to participate in pathological processes including myocardial hypertrophy, fibrosis, and apoptosis, ultimately driving HF progression [67–69]. Additionally, galectin-3 (GAL3), a β-galactoside-binding lectin, acts as a central mediator at the intersection of myocardial inflammation and fibrosis via glycan-dependent or glycan-independent mechanisms. It regulates the function of macrophages, fibroblasts, and other cells, emerging as a key driver of HF and a potential therapeutic target [70]. Meanwhile, levels of the high-molecular-weight glycoprotein antigen CA125 (cancer antigen 125; MUC16) are closely associated with fluid retention and inflammation in HF patients, serving as a reliable biomarker for assessing HF severity and prognosis [71].

With deepening understanding of glycosylation’s role in CVDs, research is gradually translating from basic mechanism exploration to clinical applications, primarily in the development of novel diagnostic biomarkers and therapeutic targets [72,73]. In the field of diagnostic biomarkers, altered levels of various circulating glycoproteins or glycan-binding proteins have been confirmed to correlate closely with the risk, severity, and prognosis of CVDs. For example, elevated von Willebrand factor is a well-recognized biomarker of thrombotic risk [74]. GAL3 and CA125 have become important biomarkers for HF diagnosis, prognostic assessment, and treatment monitoring [71,74]. Additionally, several emerging glycoprotein molecules show great potential: leucine-rich α-2-glycoprotein 1 (LRG1) and YKL-40 are associated with the development and prognosis of CVDs [75,76], while soluble SCUBE1 (signal peptide, CUB and EGF-like domain-containing protein 1) released from activated platelets holds promise as an early warning signal for acute coronary syndrome and ischemic stroke [77]. Advances in clinical glycoproteomics provide powerful tools for systematic screening and validation of glycosylation biomarkers associated with specific CVD states [72].

In terms of therapeutic targets, glycoprotein-directed interventions have become a key direction in cardiovascular drug development, particularly in antithrombotic therapy. Platelet surface glycoproteins are core targets for antithrombotic treatment [78]. Glycoprotein IIb/IIIa inhibitors, such as tirofiban, block the final common pathway of platelet aggregation and are widely used in clinical practice for acute coronary syndrome, percutaneous coronary intervention, and acute ischemic stroke, effectively preventing thrombotic events [79–82]. In recent years, platelet collagen receptor glycoprotein VI (GPVI) has been regarded as a highly promising novel antithrombotic target due to its key role in pathological thrombosis and relatively minor role in physiological hemostasis. Inhibitors targeting GPVI are expected to balance effective antithrombosis with low bleeding risk [83,84].

Beyond direct targeting of specific glycoproteins, regulating glycosylation processes themselves has emerged as an innovative therapeutic strategy. Glycoengineering technologies, such as enzymatic modification or metabolic engineering, to reprogram cell surface glycocalyx structure may be used to modulate immune responses and improve the therapeutic efficacy of CVDs [85]. Furthermore, modification of glycosylation sites in thrombolytic drugs (e.g., alteplase and tenecteplase) can optimize their pharmacokinetic properties and enhance thrombolytic efficiency and safety [86]. Targeting glycosylation signaling pathways such as *O*-GlcNAc is also considered a potential novel approach for treating diseases like ischemic stroke and HF [73]. These advances indicate that glycosylation is not only a window into understanding the pathological mechanisms of CVDs but also a promising treasure trove for drug development, opening new avenues for precise diagnosis and targeted therapy of CVDs.

## Conclusion

This study provides a comprehensive and objective overview of research progress on glycosylation in CVDs through data-driven analysis. By quantifying research trends and visualizing the results, it transforms the development of this field into measurable indicators, offering a clear and systematic representation of its evolution over time. Glycosylation, a critical PTM, encompasses various types, with O-GlcNAcylation being extensively studied for its pivotal role in CVDs. However, other glycosylation types, such as N-glycosylation and C-mannosylation, remain to be further investigated. Additionally, *Escherichia coli* may offer a novel perspective for researching on glycosylation in CVDs.

## Ethical Approval

This study did not involve human participants or animal subjects.

## Data Availability

All data are available, and source data are provided with this paper.
