# Supplementary material for: Glycosylation Profiles in Cardiovascular Diseases: A Bibliometric Analysis
Source: Health Data Sci. 2026 Feb 3;6:0409. doi: 10.34133/hds.0409 (PMC12864656; doi:10.34133/hds.0409)
Supplement: Supplementary 1 — Figs. S1 to S4 [file hds.0409.f1.zip › Supplementary.docx]

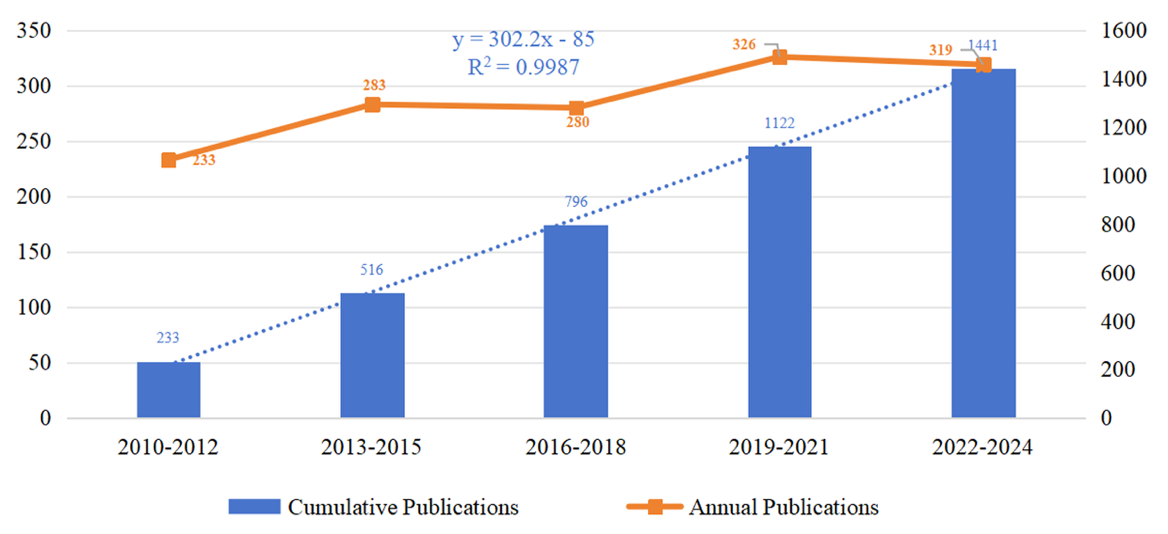


**Figure S1** Triennial and cumulative global output of research publications on glycosylation and CVDs at 3-year intervals from 2010 to 2024.


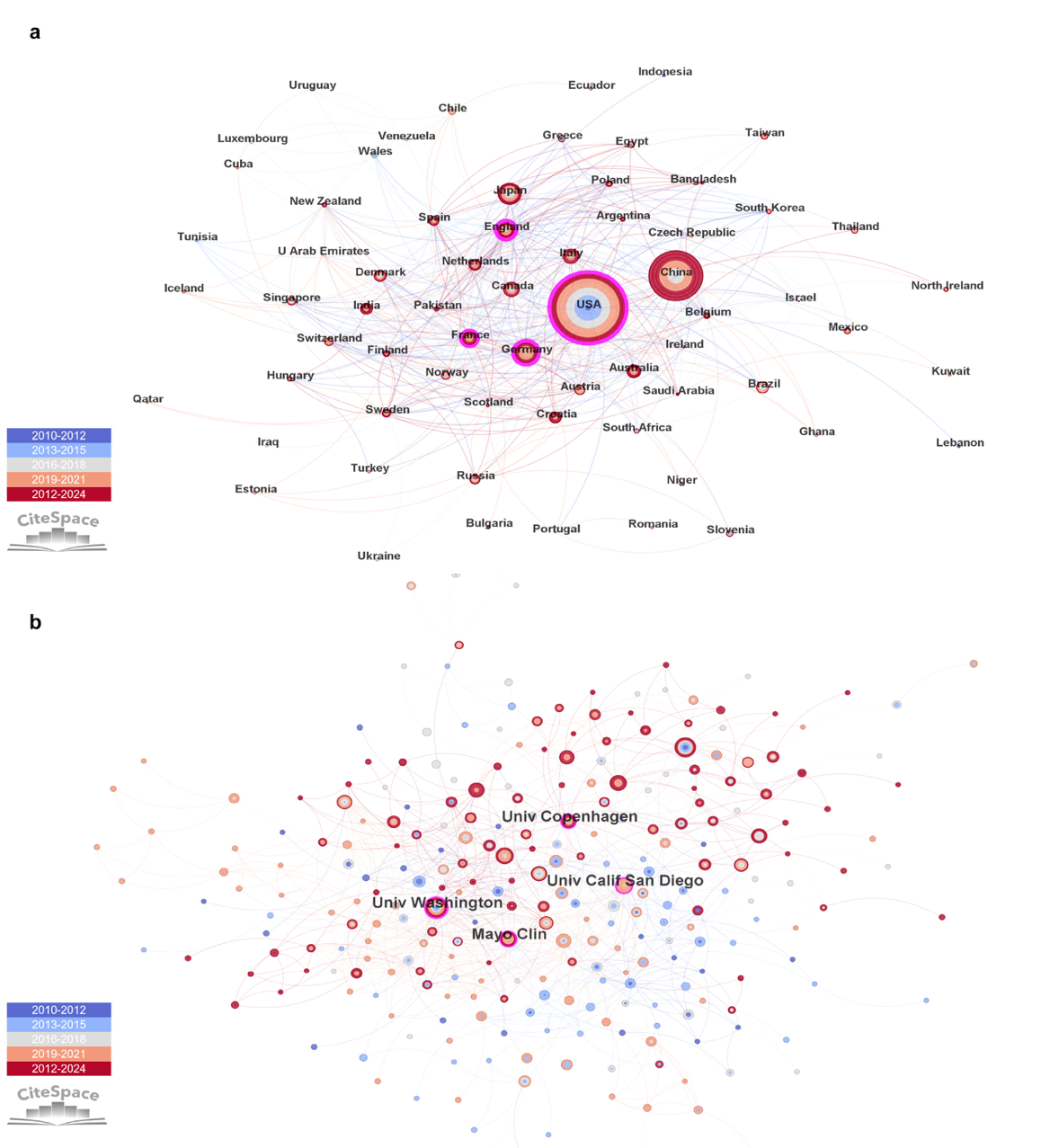


**Figure S2** (a) A network visualization map of country/regions in research for glycosylation and CVDs with triennial analysis. (b) A visual network of institutions worldwide using CiteSpace visualization with triennial analysis.


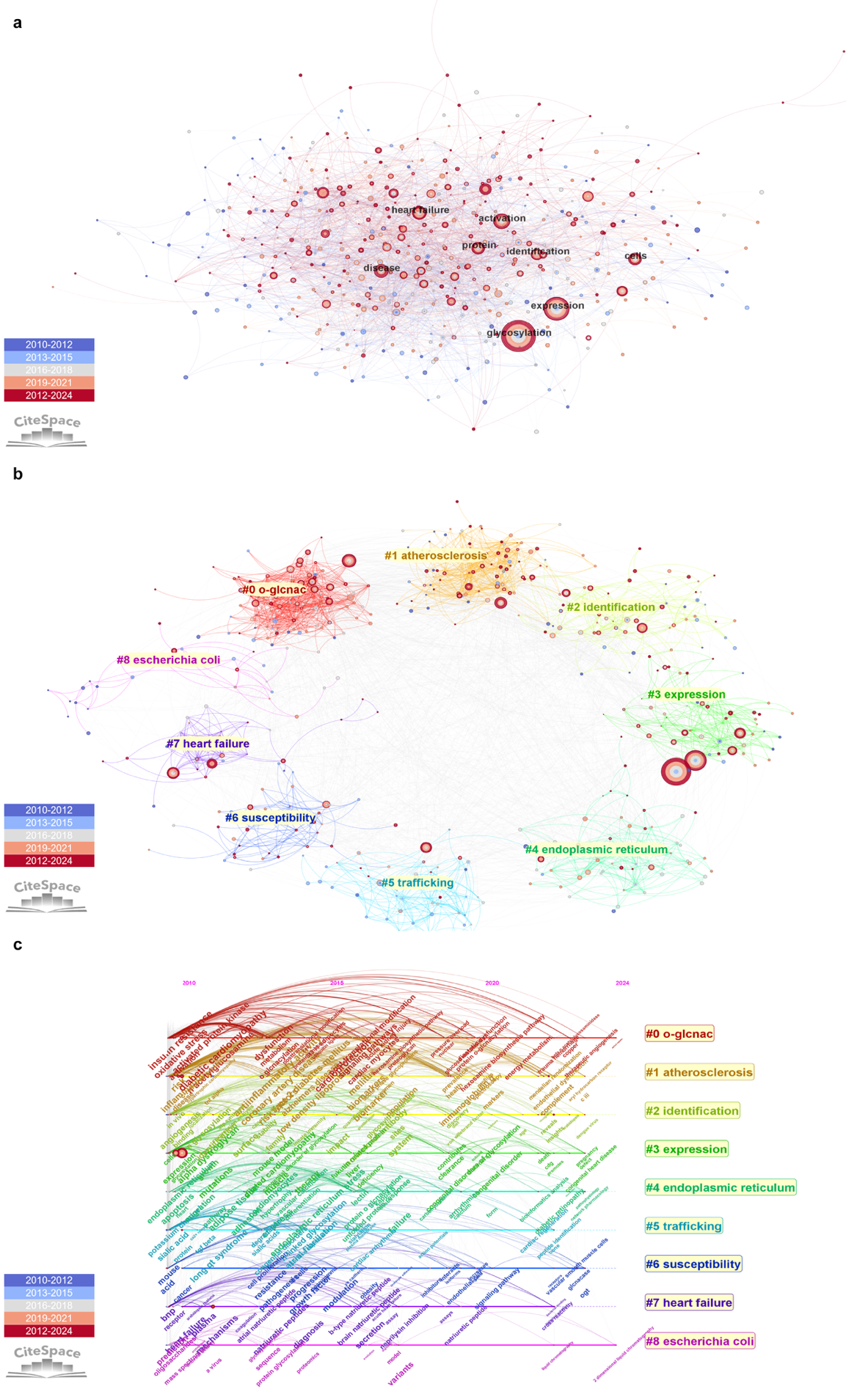


**Figure S3** Triennial analysis for keywords in the research for glycosylation and CVDs. (a) A visual network of keywords and relevant links using CiteSpace visualization. (b) The clustering map of keywords in research on glycosylation and CVDs. (c) The keyword timeline for glycosylation and CVDs.


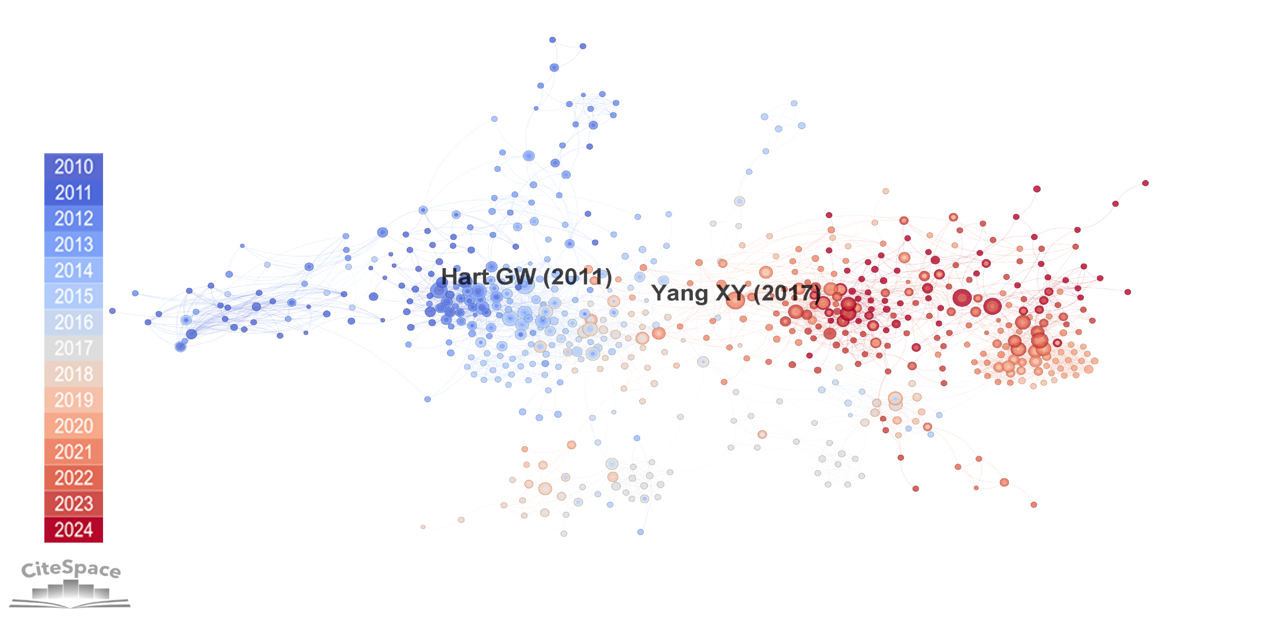


**Figure S4** A visual network of 926 references cited in the research on glycosylation and CVDs, and the top 2 references with the strongest citation bursts presented in the figure.
