# Supplementary figures and images for: Glycosylation Profiles in Cardiovascular Diseases: A Bibliometric Analysis
Source: Health Data Sci. 2026 Feb 3;6:0409. doi: 10.34133/hds.0409 (PMC12864656; doi:10.34133/hds.0409)

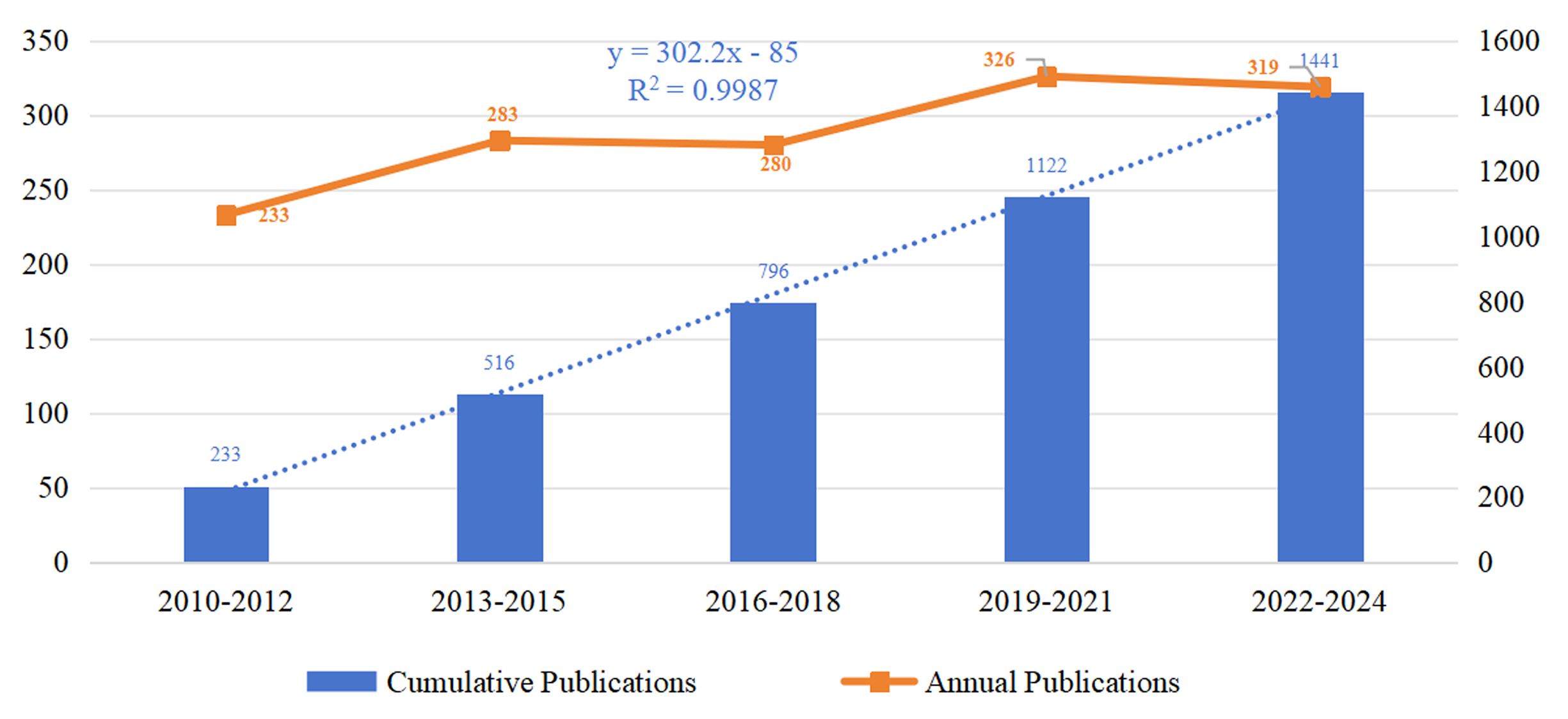

Supplement: Supplementary 1 — Figs. S1 to S4 [file hds.0409.f1.zip › Figure S1.tif]

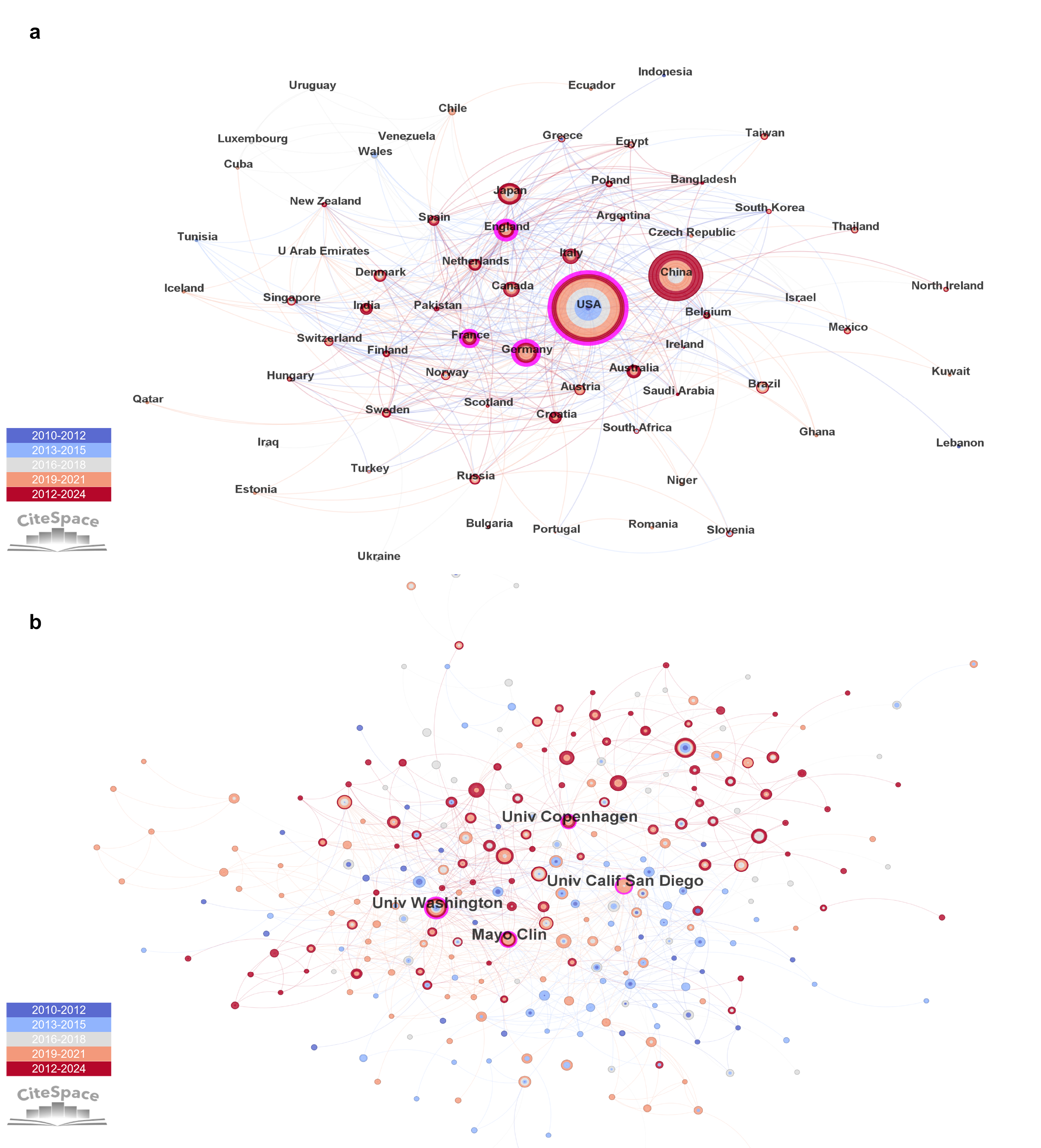

Supplement: Supplementary 1 — Figs. S1 to S4 [file hds.0409.f1.zip › Figure S2.tif]

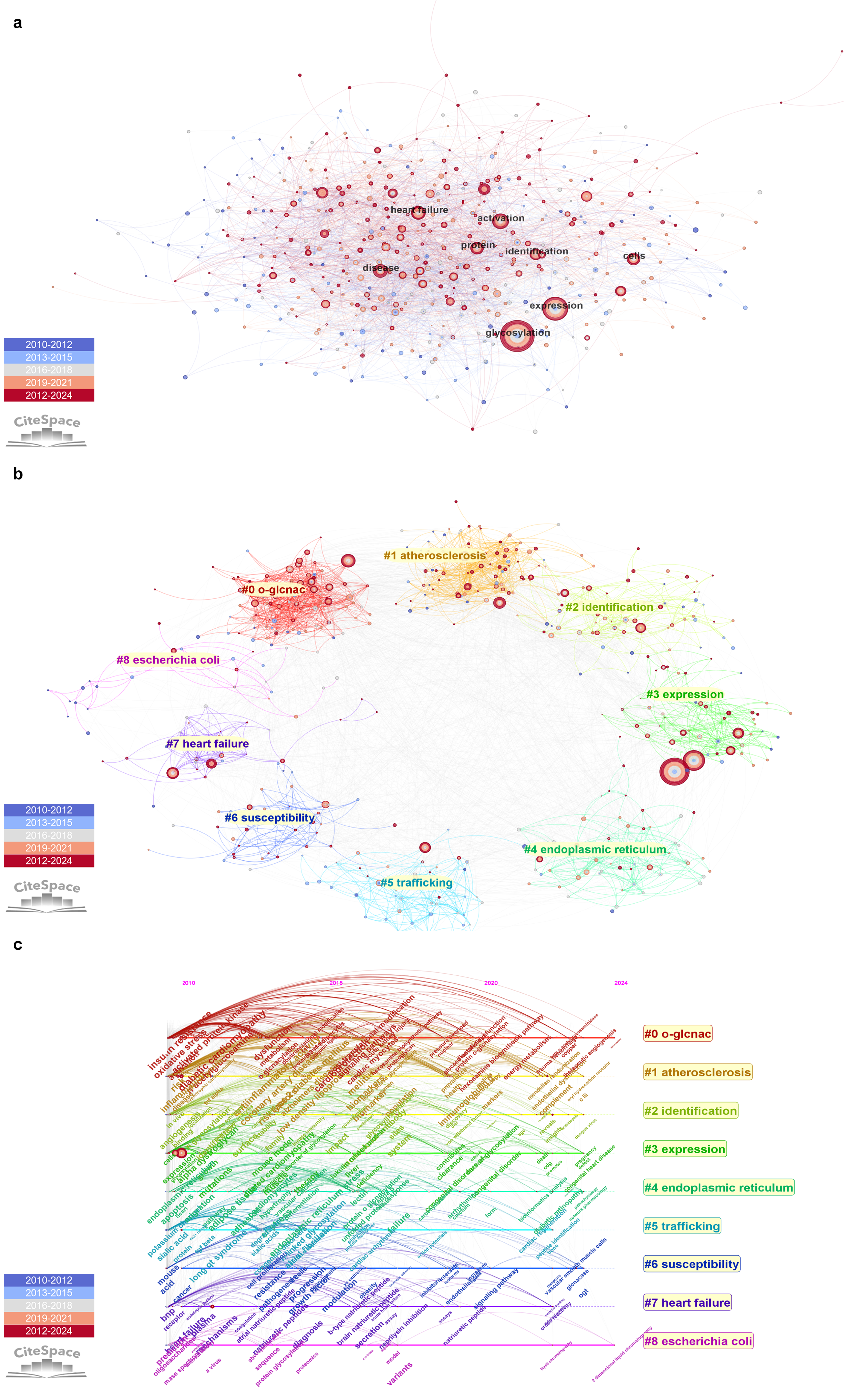

Supplement: Supplementary 1 — Figs. S1 to S4 [file hds.0409.f1.zip › Figure S3.tif]

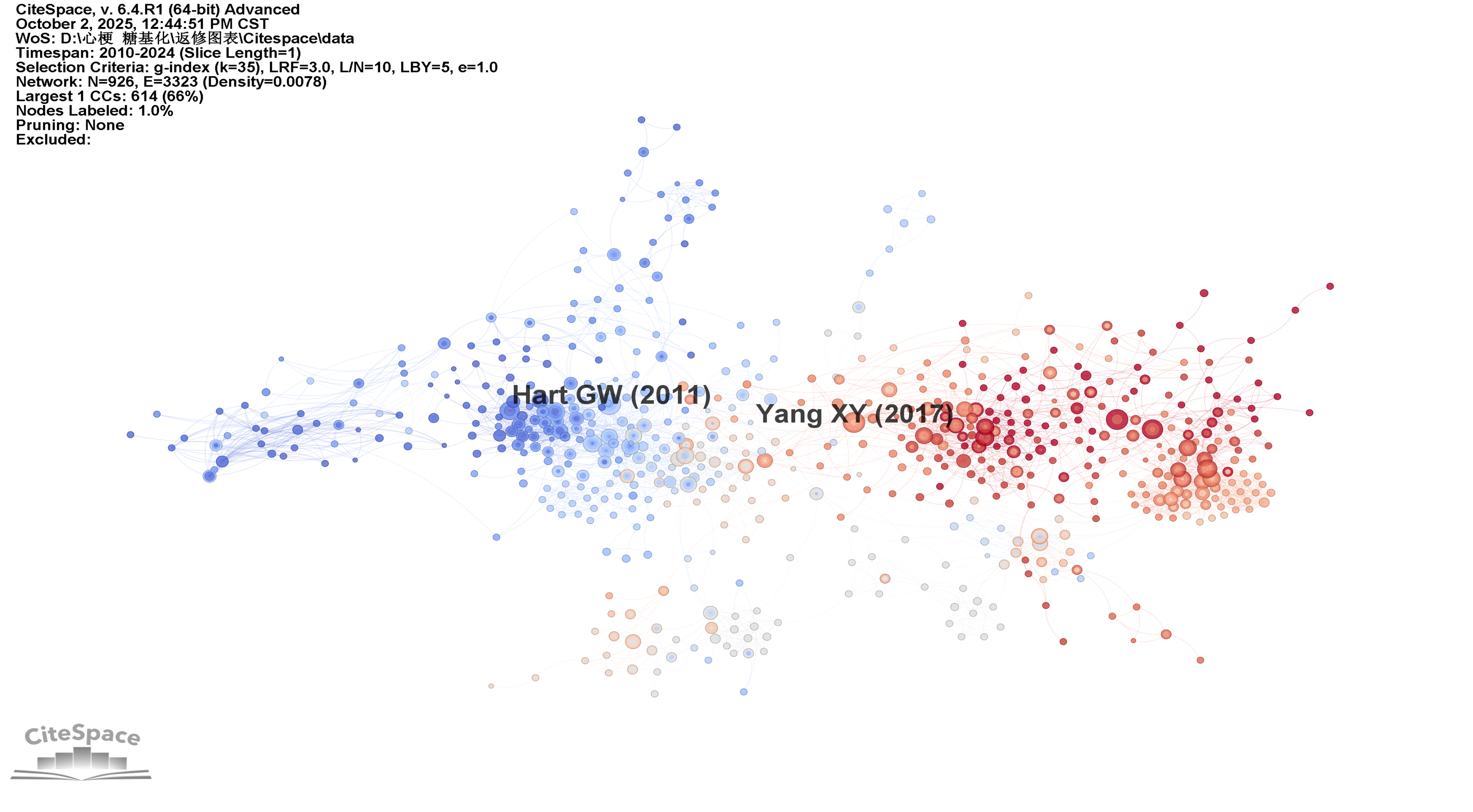

Supplement: Supplementary 1 — Figs. S1 to S4 [file hds.0409.f1.zip › Figure S4.tif]
